
JOURNAL INFORMATION
==============================
NLM Title Abbreviation: Biospektrum (Heidelb)
Journal ID: Biospektrum (Heidelb)
NLM Journal ID: 9615685

Biospektrum

ISSN: 0947-0867
EISSN: 1868-6249

ARTICLE INFORMATION
==============================
PMCID: PMC7880640
PMID: 33612987
DOI: 10.1007/s12268-021-1532-6
Article ID: 1532
Article version: 1
Subjects: Editorial

Studieren in Zeiten von Covid-19 — Chancen und Herausforderungen

Kruck Daniela kruck.daniela@mh-hannover.de info@gbm-online.de
1 2
1 grid.10423.34 0000 0000 9529 9877 Institut für Neurophysiologie, Medizinische Hochschule Hannover, Carl-Neuberg-Straße 1, D-30625 Hannover, Deutschland
2 Junior GBM c/o GBM e. V., Mörfelder Landstraße 125, D-60598 Frankfurt a. M., Deutschland
Electronic publication date: 2021 Feb 12
Print publication date: 2021
Volume: 27
Issue: 1
First page: 3
Last page: 3
Copyright: © Springer-Verlag GmbH 2021
License: Dieser Artikel wird unter der Creative Commons Namensnennung 4.0 International Lizenz veröffentlicht, welche die Nutzung, Vervielfältigung, Bearbeitung, Verbreitung und Wiedergabe in jeglichem Medium und Format erlaubt, sofern Sie den/die ursprünglichen Autor(en) und die Quelle ordnungsgemäß nennen, einen Link zur Creative Commons Lizenz beifügen und angeben, ob Änderungen vorgenommen wurden. Die in diesem Artikel enthaltenen Bilder und sonstiges Drittmaterial unterliegen ebenfalls der genannten Creative Commons Lizenz, sofern sich aus der Abbildungslegende nichts anderes ergibt. Sofern das betreffende Material nicht unter der genannten Creative Commons Lizenz steht und die betreffende Handlung nicht nach gesetzlichen Vorschriften erlaubt ist, ist für die oben aufgeführten Weiterverwendungen des Materials die Einwilligung des jeweiligen Rechteinhabers einzuholen. Weitere Details zur Lizenz entnehmen Sie bitte der Lizenzinformation auf http://creativecommons.org/licenses/by/4.0/deed.de.
License URL: https://creativecommons.org/licenses/by/4.0/

issue-copyright-statement: © Springer-Verlag GmbH Deutschland, ein Teil von Springer Nature 2021

==============================
„Nicht Nur Die Lehre Wurde In Der Pandemie Durch Ausbleiben Von Präsenzveranstaltungen Beeinflusst, Sondern Auch Das Studen Tische Leben. Die Kooperations Bereitschaft Der Fakultäten Und Digit Ale Lösungen Sind Jedoch Vorbildlich Und Sollten Auch Nach Der Pandemie Eine Wichtige Rolle Spielen“

## Studieren in Zeiten von COVID-19 – Chancen und Herausforderungen

■ Nach einem äußerst turbulenten Jahr 2020 steigt die Sehnsucht nach einer Rückkehr zu einem normalen Alltag und einem baldigen Ende der COVID-19-Pandemie. Die Folgen dieser Ausnahmesituation lassen sich in weiten Teilen der Bevölkerung spüren. Neben Arbeitnehmern diverser Berufe, Schülerinnen und Schüler sowie Kleinkindern sind auch Studierende in ganz Deutschland von der momentanen Situation betroffen. So wurde nicht nur die Lehre durch Ausbleiben von Präsenzveranstaltungen beeinflusst, auch diverse andere Aspekte des studentischen Lebens wurden geprägt. In diesem Bericht soll daher ein Blick auf die Erfahrungen der Studierenden geworfen werden, da diese Perspektive bisher wenig Aufmerksamkeit erhalten hat.

Durch die Einschränkungen im Alltag kam es bei einigen Studierenden zu einem Verlust des gewohnten Soziallebens. So wurden Interaktionen über soziale Medien und Videokonferenzen wichtiger denn je. Allerdings können solche Interaktionen den Verlust von persönlichen Treffen nur bedingt kompensieren und erleichtern nicht das Knüpfen neuer Sozialkontakte. Ein weiterer kritischer Punkt ist der Verlust von studentischen Nebenjobs. Durch die Schließung von Gastronomiebetrieben und anderen populären Arbeitgebern haben nicht nur Studierende in diesen Bereichen ihre Nebenjobs verloren. Auch studentische Hilfskräfte haben teilweise ihre Laborjobs verloren, da der vorgeschriebene Mindestabstand nicht immer eingehalten werden kann.

Aber auch innerhalb des Studiums kam es zu merklichen Einschränkungen.

Besonders das soziale Leben an den Universitäten hat sich durch die temporäre Schließung der Universitäten stark verändert. Somit ist es nicht nur schwieriger geworden, Kontakte zu anderen Studierenden zu knüpfen. Auch das Lösen von Aufgaben wurde durch die fehlende Gruppendynamik teils erschwert. Durch das Ausbleiben der Abschlussfeiern wurden Bachelor- und Masterzeugnisse postalisch verschickt, außerdem ließ sich der gelungene Studienabschluss nicht gebührend feiern.

Ein weiteres Problem war die Verschiebung von Fristen. So hat sich teilweise der Beginn der Abschlussarbeiten verzögert, da aufgrund der Maßnahmen nur eine bestimmte Zahl an Personen gleichzeitig in den Laboren arbeiten darf. Besonders kleinere Arbeitsgruppen konnten daher nicht immer einen fristgerechten Beginn garantieren. In manchen Fällen mussten Studierende sogar spontan einen neuen Platz für ihre Abschlussarbeiten in einer anderen Arbeitsgruppe finden.

Als vorbildlich wird hingegen die oftmals gelungene Umstellung auf einen Digitalunterricht gesehen. An diversen Universitäten wurden Lehrmaterial und Vorlesungen fristgerecht hochgeladen. Somit wird es Studierenden enorm erleichtert, auf Lehrmaterial zurückzugreifen und die Lehre wird insgesamt flexibler und weniger ortsgebunden.

Dies ist eine Entwicklung, über die sich einige Studierende freuen. Auch zukünftig sollte ein solches Modell mit dem alten Präsenzmodell kombiniert werden. Allerdings äußern manche Studierende ebenfalls Kritik an diesem Ansatz, da nicht alle Studierenden einen guten privaten Internetzugang bzw. einen eigenen Computer oder einen Laptop besitzen. Zudem haben nicht alle Studierenden ein ruhiges Umfeld, welches eine produktive Arbeitsatmosphäre ermöglicht.

Ein weiterer Lichtblick ist die große Hilfsbereitschaft innerhalb der Fakultäten. So konnten bspw. Fristen und Praktika flexibel geplant und – wenn nötig – verschoben werden, ohne die Studierenden zu sehr zu benachteiligen. Manche Dozenten haben den Studierenden auch mehr Infomaterial und Übungsaufgaben als zuvor bereitgestellt.

Zudem hat die Verschiebung der Veranstaltungen von Präsenzveranstaltungen zu digitalen Online-Meetings manche Studierende dazu motiviert, sich aktiver zu beteiligen. Erklärt werden kann diese Beobachtung damit, dass der Druck, vor einem großen Publikum in einem Raum zu sprechen, wegfällt, und dass durch die Chatfunktion Fragen persönlich an die Vortragenden geschickt werden können.

Insgesamt ergibt sich ein durchwachsenes Bild der momentanen Lage der Studierenden. Auch trotz guter Kooperationsbereitschaft und einer effektiven Umstellung auf einen digitalen Unterricht erleiden viele Studierende Verluste in Bezug auf das studentische Leben und können auch den Verlust von Nebenjobs und Verzögerungen von Praktika und Abschlussarbeiten nur bedingt ausgleichen. Allerdings wird die Integration von digitalen Elementen auch nach der Pandemie eine wichtige Rolle spielen, weshalb zumindest dies als positive Entwicklung bleibt. ■

**Korrespondenzadresse:**
Daniela Kruck
Institut für Neurophysiologie
Medizinische Hochschule Hannover
Carl-Neuberg-Straße 1
D-30625 Hannover
kruck.daniela@mh-hannover.de

Junior GBM c/o GBM e. V.
Mörfelder Landstraße 125
D-60598 Frankfurt a. M.
info@gbm-online.de

**Funding note:** Open Access funding enabled and organized by Projekt DEAL.
**Open Access:** Dieser Artikel wird unter der Creative Commons Namensnennung 4.0 International Lizenz veröffentlicht, welche die Nutzung, Vervielfältigung, Bearbeitung, Verbreitung und Wiedergabe in jeglichem Medium und Format erlaubt, sofern Sie den/die ursprünglichen Autor(en) und die Quelle ordnungsgemäß nennen, einen Link zur Creative Commons Lizenz beifügen und angeben, ob Änderungen vorgenommen wurden. Die in diesem Artikel enthaltenen Bilder und sonstiges Drittmaterial unterliegen ebenfalls der genannten Creative Commons Lizenz, sofern sich aus der Abbildungslegende nichts anderes ergibt. Sofern das betreffende Material nicht unter der genannten Creative Commons Lizenz steht und die betreffende Handlung nicht nach gesetzlichen Vorschriften erlaubt ist, ist für die oben aufgeführten Weiterverwendungen des Materials die Einwilligung des jeweiligen Rechteinhabers einzuholen. Weitere Details zur Lizenz entnehmen Sie bitte der Lizenzinformation auf http://creativecommons.org/licenses/by/4.0/deed.de.


Funding note

Open Access funding enabled and organized by Projekt DEAL.

